# *N*-(3,4-Dimethyl­phen­yl)-4-methyl­benzamide

**DOI:** 10.1107/S1600536809041257

**Published:** 2009-10-17

**Authors:** B. Thimme Gowda, Miroslav Tokarčík, Jozef Kožíšek, Vinola Zeena Rodrigues, Hartmut Fuess

**Affiliations:** a Department of Chemistry, Mangalore University, Mangalagangotri 574 199, Mangalore, India; b Faculty of Chemical and Food Technology, Slovak Technical University, Radlinského 9, SK-812 37 Bratislava, Slovak Republic; c Institute of Materials Science, Darmstadt University of Technology, Petersenstrasse 23, D-64287 Darmstadt, Germany

## Abstract

The title compound, C_16_H_17_NO, crystallizes with two mol­ecules in the asymmetric unit. The conformation of the N—H bond is *anti* to the *meta*-methyl substituent in the aniline ring in the first mol­ecule and *syn* in the second mol­ecule. The dihedral angles between the two benzene rings are 52.6 (1) and 10.5 (1)° in the first and second mol­ecules, respectively. Inter­molecular N—H⋯O hydrogen bonds link the mol­ecules into chains running along the *b* axis of the crystal.

## Related literature

For the preparation of the title compound, see: Gowda *et al.* (2003 ▶). For related structures, see: Bowes *et al.* (2003 ▶); Gowda *et al.* (2008 ▶, 2009 ▶).
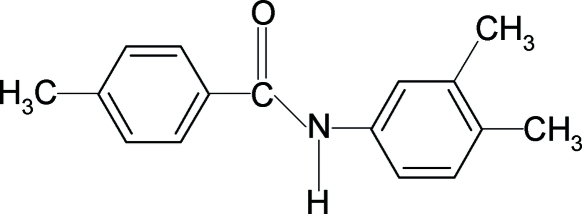


## Experimental

### 

#### Crystal data

C_16_H_17_NO
*M* *_r_* = 239.31
Triclinic, 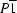

*a* = 9.4186 (3) Å
*b* = 9.55915 (18) Å
*c* = 15.8813 (4) Å
α = 74.361 (2)°
β = 79.696 (2)°
γ = 88.1582 (18)°
*V* = 1354.51 (6) Å^3^
*Z* = 4
Mo *K*α radiation
μ = 0.07 mm^−1^
*T* = 295 K
0.51 × 0.41 × 0.22 mm

#### Data collection

Oxford Diffraction Xcalibur diffractometer with a Ruby Gemini detector
Absorption correction: multi-scan (*CrysAlis Pro*; Oxford Diffraction, 2009 ▶) *T*_min_ = 0.943, *T*_max_ = 0.981
24655 measured reflections
5130 independent reflections
3880 reflections with *I* > 2σ(*I*)
*R*_int_ = 0.016

#### Refinement

*R*[*F* ^2^ > 2σ(*F* ^2^)] = 0.042
*wR*(*F* ^2^) = 0.126
*S* = 1.09
5130 reflections
332 parameters
2 restraints
H-atom parameters constrained
Δρ_max_ = 0.19 e Å^−3^
Δρ_min_ = −0.13 e Å^−3^

### 

Data collection: *CrysAlis Pro* (Oxford Diffraction, 2009 ▶); cell refinement: *CrysAlis Pro*; data reduction: *CrysAlis Pro*; program(s) used to solve structure: *SHELXS97* (Sheldrick, 2008 ▶); program(s) used to refine structure: *SHELXL97* (Sheldrick, 2008 ▶); molecular graphics: *ORTEP-3* (Farrugia, 1997 ▶) and *DIAMOND* (Brandenburg, 2002 ▶); software used to prepare material for publication: *SHELXL97*, *PLATON* (Spek, 2009 ▶) and *WinGX* (Farrugia, 1999 ▶).

## Supplementary Material

Crystal structure: contains datablocks I, global. DOI: 10.1107/S1600536809041257/bt5089sup1.cif

Structure factors: contains datablocks I. DOI: 10.1107/S1600536809041257/bt5089Isup2.hkl

Additional supplementary materials:  crystallographic information; 3D view; checkCIF report

## Figures and Tables

**Table 1:** Hydrogen-bond geometry (Å, °)

*D*—H⋯*A*	*D*—H	H⋯*A*	*D*⋯*A*	*D*—H⋯*A*
N1—H1*N*⋯O2^i^	0.86	2.29	3.0860 (14)	154
N2—H2*N*⋯O1^ii^	0.86	2.18	3.0101 (14)	162

Symmetry codes: (i) 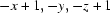; (ii) 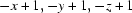.

**Fig. 1.**
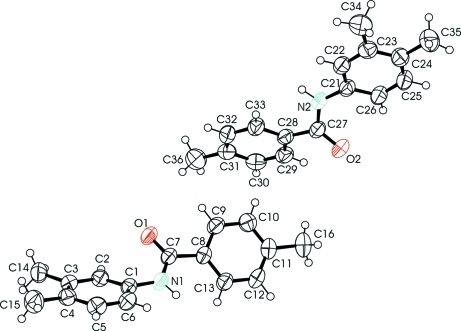
Molecular structure of of the title compound showing the atom labelling scheme. Displacement ellipsoids are drawn at the 50% probability level. H atoms are represented as small spheres of arbitrary radii.

**Fig. 2.**
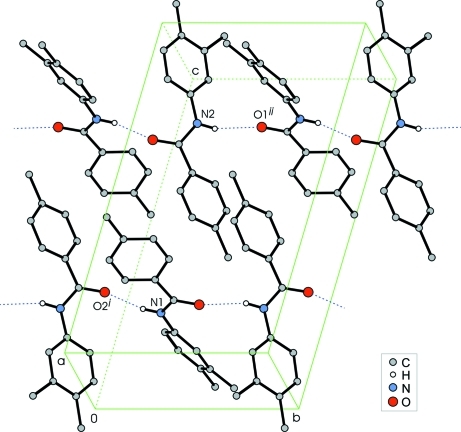
Part of the crystal structure of the title compound. Molecular chains running along the b-axis are generated by N–H···O hydrogen bonds, shown as dashed lines. Symmetry codes (i): -x + 1,-y,-z + 1; (ii) -x + 1,-y + 1,-z + 1. H atoms not involved in hydrogen bonding are omitted.

## supplementary crystallographic information

### Comment

As part of a study of the substituent effects on the crystal structures of benzanilides (Gowda, Tokarčík *et al.*, 2008, 2009), the structure of *N*-(3,4-dimethylphenyl)4-methylbenzamide (I) has been determined. In the structure, the conformations of the N—H and C=O bonds are *anti* to each other (Fig. 1), similar to those observed in *N*-(3,4-dimethylphenyl)benzamide (Gowda, Tokarčík *et al.*, 2008), *N*-(2,6-dimethylphenyl)4-methylbenzamide (Gowda, Tokarčík *et al.*, 2009) and the parent benzanilide (Bowes *et al.*, 2003).

The asymmetric unit of the cell in (I) contains two independent molecules. In the first molecule, the conformation of the N—H bond is *anti* to the *meta*-methyl-substituent in the disubstituted phenyl ring, while in the second molecule this conformation is *syn*. The dihedral angles between the two benzene rings are 52.6 (1)° and 10.5 (1)° in the first and second molecules, respectively. The central amido group –NH—C(=O)- forms dihedral angles of 22.2 (2)° and 31.2 (1)° with the benzoyl ring, and 30.6 (1)° and 25.5 (1)° with the disubstituted phenyl ring, in the two independent molecules.

The packing diagram of molecules in (I) showing the intermolecular N–H···O hydrogen bonds (Table 1) involved in the formation of molecular chains running along the *b*-axis is shown in Fig. 2.

### Experimental

The title compound was prepared according to the literature method (Gowda *et al.*, 2003). The purity of the compound was checked by determining its melting point. It was characterized by recording its infrared and NMR spectra. Single crystals of the title compound used in X-ray diffraction studies were obtained from a slow evaporation of its ethanolic solution at room temperature.

### Refinement

H atoms were placed in calculated positions with N–H distances of 0.86 Å and C–H distances in the range 0.93–0.96 Å. All hydrogen atoms were constrained to ride on their parent atoms. The *U*_iso_(H) values were set at 1.2*U*_eq_(C aromatic, N) and 1.5 *U*_eq_(C_methyl_). The *U* values of the atom pairs C23—C34 and C7—O1 were subject to a rigid bond restraint (DELU instruction), *i.e.* the components of the displacement parameters in the direction of the bond were restrained to be equal within an effective standard deviation 0.004. The C16 and C36 methyl group exhibit orientational disorder in the hydrogen atom positions. The two sets of methyl hydrogen atoms were refined with equal occupancy.

### Crystal data

**Table:**

C_16_H_17_NO	*Z* = 4
*M**_r_* = 239.31	*F*(000) = 512
Triclinic, *P*1	*D*_x_ = 1.173 Mg m^−^^3^
Hall symbol: -P 1	Mo *K*α radiation, λ = 0.71073 Å
*a* = 9.4186 (3) Å	Cell parameters from 13000 reflections
*b* = 9.55915 (18) Å	θ = 2.2–29.3°
*c* = 15.8813 (4) Å	µ = 0.07 mm^−^^1^
α = 74.361 (2)°	*T* = 295 K
β = 79.696 (2)°	Block, colourless
γ = 88.1582 (18)°	0.51 × 0.41 × 0.22 mm
*V* = 1354.51 (6) Å^3^	

### Data collection

**Table:**

Oxford Diffraction Xcalibur with a Ruby Gemini detector diffractometer	5130 independent reflections
graphite	3880 reflections with *I* > 2σ(*I*)
Detector resolution: 10.434 pixels mm^-1^	*R*_int_ = 0.016
ω scans	θ_max_ = 25.7°, θ_min_ = 2.2°
Absorption correction: multi-scan (*CrysAlis PRO*; Oxford Diffraction, 2009)	*h* = −11→11
*T*_min_ = 0.943, *T*_max_ = 0.981	*k* = −11→11
24655 measured reflections	*l* = −19→19

### Refinement

**Table:**

Refinement on *F*^2^	Primary atom site location: structure-invariant direct methods
Least-squares matrix: full	Secondary atom site location: difference Fourier map
*R*[*F*^2^ > 2σ(*F*^2^)] = 0.042	Hydrogen site location: inferred from neighbouring sites
*wR*(*F*^2^) = 0.126	H-atom parameters constrained
*S* = 1.09	*w* = 1/[σ^2^(*F*_o_^2^) + (0.0705*P*)^2^ + 0.0954*P*] where *P* = (*F*_o_^2^ + 2*F*_c_^2^)/3
5130 reflections	(Δ/σ)_max_ < 0.001
332 parameters	Δρ_max_ = 0.19 e Å^−^^3^
2 restraints	Δρ_min_ = −0.13 e Å^−^^3^

### Special details

**Table:**

Geometry. All e.s.d.'s (except the e.s.d. in the dihedral angle between two l.s. planes) are estimated using the full covariance matrix. The cell e.s.d.'s are taken into account individually in the estimation of e.s.d.'s in distances, angles and torsion angles; correlations between e.s.d.'s in cell parameters are only used when they are defined by crystal symmetry. An approximate (isotropic) treatment of cell e.s.d.'s is used for estimating e.s.d.'s involving l.s. planes.
Refinement. Refinement of *F*^2^ against ALL reflections. The weighted *R*-factor *wR* and goodness of fit *S* are based on *F*^2^, conventional *R*-factors *R* are based on *F*, with *F* set to zero for negative *F*^2^. The threshold expression of *F*^2^ > σ(*F*^2^) is used only for calculating *R*-factors(gt) *etc*. and is not relevant to the choice of reflections for refinement. *R*-factors based on *F*^2^ are statistically about twice as large as those based on *F*, and *R*- factors based on ALL data will be even larger.

### Fractional atomic coordinates and isotropic or equivalent isotropic displacement parameters (Å^2^)

**Table:**

	*x*	*y*	*z*	*U*_iso_*/*U*_eq_	Occ. (<1)
C1	0.10346 (14)	0.30182 (13)	0.21469 (9)	0.0465 (3)	
C2	0.11199 (14)	0.43910 (14)	0.15658 (9)	0.0488 (3)	
H2	0.1996	0.4899	0.1399	0.059*	
C3	−0.00845 (15)	0.50305 (14)	0.12241 (9)	0.0502 (3)	
C4	−0.13954 (15)	0.42676 (15)	0.14676 (10)	0.0536 (4)	
C5	−0.14410 (15)	0.28750 (16)	0.20304 (10)	0.0571 (4)	
H5	−0.2303	0.2345	0.2184	0.069*	
C6	−0.02543 (15)	0.22486 (15)	0.23707 (10)	0.0538 (4)	
H6	−0.0322	0.1313	0.2749	0.065*	
C7	0.32980 (14)	0.30304 (13)	0.27355 (9)	0.0483 (3)	
C8	0.43956 (14)	0.20801 (13)	0.31706 (9)	0.0464 (3)	
C9	0.51877 (16)	0.26395 (15)	0.36612 (10)	0.0569 (4)	
H9	0.5038	0.3591	0.3695	0.068*	
C10	0.61908 (16)	0.18210 (16)	0.40995 (10)	0.0601 (4)	
H10	0.6691	0.2219	0.4437	0.072*	
C11	0.64706 (15)	0.04163 (15)	0.40481 (10)	0.0549 (4)	
C12	0.57052 (16)	−0.01379 (15)	0.35434 (11)	0.0603 (4)	
H12	0.5887	−0.1076	0.3491	0.072*	
C13	0.46711 (16)	0.06721 (14)	0.31119 (10)	0.0554 (4)	
H13	0.416	0.027	0.2782	0.067*	
C14	0.00537 (19)	0.65361 (16)	0.06181 (11)	0.0678 (4)	
H14A	0.1056	0.6805	0.0424	0.102*	
H14B	−0.0423	0.7201	0.0929	0.102*	
H14C	−0.0384	0.6569	0.0112	0.102*	
C15	−0.27382 (18)	0.4940 (2)	0.11380 (14)	0.0790 (5)	
H15A	−0.287	0.5882	0.1243	0.118*	
H15B	−0.3562	0.433	0.1449	0.118*	
H15C	−0.2636	0.5034	0.0513	0.118*	
C16	0.75981 (18)	−0.04604 (19)	0.45196 (12)	0.0738 (5)	
H16A	0.819	0.0174	0.4694	0.111*	0.5
H16B	0.819	−0.094	0.4127	0.111*	0.5
H16C	0.7132	−0.1173	0.5038	0.111*	0.5
H16D	0.7484	−0.1466	0.4546	0.111*	0.5
H16E	0.7484	−0.0353	0.5112	0.111*	0.5
H16F	0.8542	−0.012	0.4201	0.111*	0.5
N1	0.22333 (12)	0.23479 (11)	0.25316 (8)	0.0522 (3)	
H1N	0.2277	0.1417	0.2643	0.063*	
O1	0.33726 (11)	0.43667 (9)	0.25845 (7)	0.0638 (3)	
C21	0.74411 (15)	0.18251 (13)	0.82751 (10)	0.0510 (3)	
C22	0.64037 (16)	0.22586 (15)	0.88897 (10)	0.0549 (4)	
H22	0.5795	0.3012	0.8688	0.066*	
C23	0.62458 (16)	0.16037 (16)	0.97944 (10)	0.0585 (4)	
C24	0.71696 (18)	0.04716 (15)	1.00955 (10)	0.0626 (4)	
C25	0.81944 (19)	0.00514 (16)	0.94732 (11)	0.0651 (4)	
H25	0.8804	−0.0704	0.9671	0.078*	
C26	0.83512 (17)	0.07014 (15)	0.85765 (11)	0.0605 (4)	
H26	0.9056	0.0393	0.8178	0.073*	
C27	0.81041 (15)	0.21391 (14)	0.66478 (10)	0.0513 (3)	
C28	0.81065 (15)	0.32107 (14)	0.57693 (9)	0.0490 (3)	
C29	0.91799 (16)	0.31421 (16)	0.50555 (11)	0.0575 (4)	
H29	0.987	0.2423	0.5134	0.069*	
C30	0.92379 (17)	0.41186 (17)	0.42359 (11)	0.0621 (4)	
H30	0.9981	0.4065	0.3774	0.074*	
C31	0.82068 (18)	0.51821 (16)	0.40875 (10)	0.0598 (4)	
C32	0.71185 (18)	0.52273 (16)	0.47904 (10)	0.0612 (4)	
H32	0.6403	0.5918	0.4703	0.073*	
C33	0.70702 (16)	0.42699 (15)	0.56190 (10)	0.0567 (4)	
H33	0.6334	0.4335	0.6082	0.068*	
C34	0.5104 (2)	0.2114 (2)	1.04295 (12)	0.0811 (5)	
H34A	0.5544	0.2391	1.0863	0.122*	
H34B	0.4413	0.1343	1.0724	0.122*	
H34C	0.4627	0.2934	1.0108	0.122*	
C35	0.7065 (2)	−0.0265 (2)	1.10752 (12)	0.0877 (6)	
H35A	0.7735	−0.1047	1.1154	0.132*	
H35B	0.6102	−0.0641	1.1319	0.132*	
H35C	0.729	0.0427	1.1374	0.132*	
C36	0.8276 (2)	0.6250 (2)	0.31868 (12)	0.0885 (6)	
H36A	0.9105	0.6056	0.2788	0.133*	0.5
H36B	0.8349	0.722	0.324	0.133*	0.5
H36C	0.7418	0.6152	0.2959	0.133*	0.5
H36D	0.7476	0.6896	0.3203	0.133*	0.5
H36E	0.8232	0.5732	0.2751	0.133*	0.5
H36F	0.9164	0.68	0.3033	0.133*	0.5
N2	0.75630 (13)	0.26110 (11)	0.73672 (8)	0.0549 (3)	
H2N	0.7257	0.3488	0.7264	0.066*	
O2	0.85769 (12)	0.09069 (10)	0.67005 (7)	0.0667 (3)	

### Atomic displacement parameters (Å^2^)

**Table:**

	*U*^11^	*U*^22^	*U*^33^	*U*^12^	*U*^13^	*U*^23^
C1	0.0471 (7)	0.0423 (7)	0.0512 (8)	0.0065 (6)	−0.0090 (6)	−0.0149 (6)
C2	0.0438 (7)	0.0467 (7)	0.0571 (8)	−0.0020 (6)	−0.0106 (6)	−0.0144 (6)
C3	0.0564 (8)	0.0446 (7)	0.0529 (8)	0.0033 (6)	−0.0142 (7)	−0.0158 (6)
C4	0.0478 (8)	0.0568 (8)	0.0618 (9)	0.0048 (6)	−0.0153 (7)	−0.0223 (7)
C5	0.0457 (8)	0.0577 (8)	0.0681 (10)	−0.0055 (7)	−0.0070 (7)	−0.0183 (7)
C6	0.0537 (8)	0.0452 (7)	0.0590 (9)	−0.0006 (6)	−0.0042 (7)	−0.0115 (6)
C7	0.0518 (8)	0.0403 (6)	0.0518 (8)	0.0060 (6)	−0.0073 (6)	−0.0123 (6)
C8	0.0447 (7)	0.0406 (7)	0.0491 (8)	0.0056 (6)	−0.0035 (6)	−0.0078 (6)
C9	0.0590 (9)	0.0468 (7)	0.0689 (10)	0.0088 (7)	−0.0138 (7)	−0.0215 (7)
C10	0.0569 (9)	0.0615 (9)	0.0638 (10)	0.0040 (7)	−0.0158 (7)	−0.0171 (7)
C11	0.0454 (8)	0.0547 (8)	0.0542 (8)	0.0054 (6)	−0.0045 (6)	−0.0004 (7)
C12	0.0628 (9)	0.0410 (7)	0.0742 (10)	0.0126 (7)	−0.0124 (8)	−0.0114 (7)
C13	0.0561 (8)	0.0461 (7)	0.0661 (9)	0.0083 (6)	−0.0155 (7)	−0.0162 (7)
C14	0.0778 (11)	0.0526 (8)	0.0736 (11)	0.0044 (8)	−0.0314 (9)	−0.0069 (8)
C15	0.0593 (10)	0.0817 (11)	0.1008 (14)	0.0088 (9)	−0.0308 (9)	−0.0226 (10)
C16	0.0613 (10)	0.0733 (10)	0.0743 (11)	0.0121 (8)	−0.0174 (8)	0.0035 (9)
N1	0.0549 (7)	0.0350 (5)	0.0658 (8)	0.0044 (5)	−0.0144 (6)	−0.0101 (5)
O1	0.0730 (7)	0.0377 (4)	0.0875 (8)	0.0087 (5)	−0.0319 (6)	−0.0175 (5)
C21	0.0560 (8)	0.0375 (6)	0.0605 (9)	−0.0002 (6)	−0.0165 (7)	−0.0106 (6)
C22	0.0542 (8)	0.0464 (7)	0.0659 (10)	0.0028 (6)	−0.0163 (7)	−0.0148 (7)
C23	0.0608 (9)	0.0524 (8)	0.0644 (10)	−0.0087 (7)	−0.0128 (7)	−0.0168 (7)
C24	0.0765 (10)	0.0475 (8)	0.0630 (10)	−0.0104 (7)	−0.0211 (8)	−0.0063 (7)
C25	0.0776 (11)	0.0451 (8)	0.0720 (11)	0.0081 (7)	−0.0262 (9)	−0.0072 (7)
C26	0.0673 (10)	0.0451 (7)	0.0689 (10)	0.0092 (7)	−0.0175 (8)	−0.0124 (7)
C27	0.0501 (8)	0.0401 (7)	0.0668 (9)	0.0054 (6)	−0.0163 (7)	−0.0163 (6)
C28	0.0500 (8)	0.0417 (7)	0.0604 (9)	0.0033 (6)	−0.0162 (7)	−0.0185 (6)
C29	0.0523 (8)	0.0555 (8)	0.0726 (10)	0.0107 (7)	−0.0161 (7)	−0.0287 (8)
C30	0.0642 (10)	0.0676 (9)	0.0596 (9)	−0.0015 (8)	−0.0054 (8)	−0.0291 (8)
C31	0.0743 (10)	0.0550 (8)	0.0552 (9)	0.0011 (7)	−0.0147 (8)	−0.0215 (7)
C32	0.0705 (10)	0.0540 (8)	0.0600 (9)	0.0164 (7)	−0.0172 (8)	−0.0152 (7)
C33	0.0595 (9)	0.0520 (8)	0.0577 (9)	0.0122 (7)	−0.0082 (7)	−0.0157 (7)
C34	0.0821 (12)	0.0884 (12)	0.0752 (12)	−0.0012 (10)	−0.0092 (9)	−0.0289 (10)
C35	0.1162 (16)	0.0751 (11)	0.0675 (11)	−0.0026 (11)	−0.0260 (11)	−0.0052 (9)
C36	0.1194 (16)	0.0842 (12)	0.0585 (11)	0.0085 (11)	−0.0133 (10)	−0.0158 (9)
N2	0.0663 (8)	0.0392 (6)	0.0596 (7)	0.0121 (5)	−0.0157 (6)	−0.0123 (5)
O2	0.0815 (7)	0.0407 (5)	0.0802 (7)	0.0160 (5)	−0.0193 (6)	−0.0187 (5)

### Geometric parameters (Å, °)

**Table:**

C1—C6	1.3796 (19)	C21—C26	1.3885 (19)
C1—C2	1.3812 (18)	C21—C22	1.389 (2)
C1—N1	1.4309 (16)	C21—N2	1.4209 (18)
C2—C3	1.3970 (18)	C22—C23	1.387 (2)
C2—H2	0.93	C22—H22	0.93
C3—C4	1.393 (2)	C23—C24	1.402 (2)
C3—C14	1.495 (2)	C23—C34	1.501 (2)
C4—C5	1.386 (2)	C24—C25	1.385 (2)
C4—C15	1.513 (2)	C24—C35	1.512 (2)
C5—C6	1.378 (2)	C25—C26	1.375 (2)
C5—H5	0.93	C25—H25	0.93
C6—H6	0.93	C26—H26	0.93
C7—O1	1.2364 (15)	C27—O2	1.2326 (15)
C7—N1	1.3448 (17)	C27—N2	1.3502 (18)
C7—C8	1.4927 (18)	C27—C28	1.491 (2)
C8—C9	1.3812 (19)	C28—C33	1.3872 (19)
C8—C13	1.3871 (18)	C28—C29	1.392 (2)
C9—C10	1.372 (2)	C29—C30	1.375 (2)
C9—H9	0.93	C29—H29	0.93
C10—C11	1.381 (2)	C30—C31	1.386 (2)
C10—H10	0.93	C30—H30	0.93
C11—C12	1.380 (2)	C31—C32	1.383 (2)
C11—C16	1.510 (2)	C31—C36	1.508 (2)
C12—C13	1.3863 (19)	C32—C33	1.380 (2)
C12—H12	0.93	C32—H32	0.93
C13—H13	0.93	C33—H33	0.93
C14—H14A	0.96	C34—H34A	0.96
C14—H14B	0.96	C34—H34B	0.96
C14—H14C	0.96	C34—H34C	0.96
C15—H15A	0.96	C35—H35A	0.96
C15—H15B	0.96	C35—H35B	0.96
C15—H15C	0.96	C35—H35C	0.96
C16—H16A	0.96	C36—H36A	0.96
C16—H16B	0.96	C36—H36B	0.96
C16—H16C	0.96	C36—H36C	0.96
C16—H16D	0.96	C36—H36D	0.96
C16—H16E	0.96	C36—H36E	0.96
C16—H16F	0.96	C36—H36F	0.96
N1—H1N	0.86	N2—H2N	0.86
			
C6—C1—C2	119.20 (12)	C26—C21—C22	118.95 (14)
C6—C1—N1	118.01 (11)	C26—C21—N2	123.14 (13)
C2—C1—N1	122.79 (12)	C22—C21—N2	117.83 (12)
C1—C2—C3	121.25 (12)	C23—C22—C21	122.13 (13)
C1—C2—H2	119.4	C23—C22—H22	118.9
C3—C2—H2	119.4	C21—C22—H22	118.9
C4—C3—C2	119.52 (12)	C22—C23—C24	118.70 (14)
C4—C3—C14	121.29 (13)	C22—C23—C34	119.93 (14)
C2—C3—C14	119.17 (13)	C24—C23—C34	121.37 (15)
C5—C4—C3	118.06 (12)	C25—C24—C23	118.36 (14)
C5—C4—C15	120.72 (14)	C25—C24—C35	120.60 (15)
C3—C4—C15	121.22 (13)	C23—C24—C35	121.03 (16)
C6—C5—C4	122.34 (13)	C26—C25—C24	122.92 (14)
C6—C5—H5	118.8	C26—C25—H25	118.5
C4—C5—H5	118.8	C24—C25—H25	118.5
C5—C6—C1	119.57 (13)	C25—C26—C21	118.94 (15)
C5—C6—H6	120.2	C25—C26—H26	120.5
C1—C6—H6	120.2	C21—C26—H26	120.5
O1—C7—N1	122.74 (12)	O2—C27—N2	122.99 (13)
O1—C7—C8	121.06 (12)	O2—C27—C28	121.21 (13)
N1—C7—C8	116.20 (11)	N2—C27—C28	115.80 (11)
C9—C8—C13	117.98 (12)	C33—C28—C29	117.72 (13)
C9—C8—C7	117.90 (11)	C33—C28—C27	123.19 (13)
C13—C8—C7	124.11 (12)	C29—C28—C27	119.08 (12)
C10—C9—C8	121.37 (13)	C30—C29—C28	121.18 (13)
C10—C9—H9	119.3	C30—C29—H29	119.4
C8—C9—H9	119.3	C28—C29—H29	119.4
C9—C10—C11	121.13 (14)	C29—C30—C31	121.05 (14)
C9—C10—H10	119.4	C29—C30—H30	119.5
C11—C10—H10	119.4	C31—C30—H30	119.5
C12—C11—C10	117.77 (13)	C32—C31—C30	117.79 (14)
C12—C11—C16	121.72 (14)	C32—C31—C36	121.41 (15)
C10—C11—C16	120.50 (15)	C30—C31—C36	120.79 (15)
C11—C12—C13	121.47 (13)	C33—C32—C31	121.46 (14)
C11—C12—H12	119.3	C33—C32—H32	119.3
C13—C12—H12	119.3	C31—C32—H32	119.3
C12—C13—C8	120.26 (13)	C32—C33—C28	120.77 (14)
C12—C13—H13	119.9	C32—C33—H33	119.6
C8—C13—H13	119.9	C28—C33—H33	119.6
C3—C14—H14A	109.5	C23—C34—H34A	109.5
C3—C14—H14B	109.5	C23—C34—H34B	109.5
H14A—C14—H14B	109.5	H34A—C34—H34B	109.5
C3—C14—H14C	109.5	C23—C34—H34C	109.5
H14A—C14—H14C	109.5	H34A—C34—H34C	109.5
H14B—C14—H14C	109.5	H34B—C34—H34C	109.5
C4—C15—H15A	109.5	C24—C35—H35A	109.5
C4—C15—H15B	109.5	C24—C35—H35B	109.5
H15A—C15—H15B	109.5	H35A—C35—H35B	109.5
C4—C15—H15C	109.5	C24—C35—H35C	109.5
H15A—C15—H15C	109.5	H35A—C35—H35C	109.5
H15B—C15—H15C	109.5	H35B—C35—H35C	109.5
C11—C16—H16A	109.5	C31—C36—H36A	109.5
C11—C16—H16B	109.5	C31—C36—H36B	109.5
H16A—C16—H16B	109.5	H36A—C36—H36B	109.5
C11—C16—H16C	109.5	C31—C36—H36C	109.5
H16A—C16—H16C	109.5	H36A—C36—H36C	109.5
H16B—C16—H16C	109.5	H36B—C36—H36C	109.5
C11—C16—H16D	109.5	C31—C36—H36D	109.5
H16A—C16—H16D	141.1	H36A—C36—H36D	141.1
H16B—C16—H16D	56.3	H36B—C36—H36D	56.3
H16C—C16—H16D	56.3	H36C—C36—H36D	56.3
C11—C16—H16E	109.5	C31—C36—H36E	109.5
H16A—C16—H16E	56.3	H36A—C36—H36E	56.3
H16B—C16—H16E	141.1	H36B—C36—H36E	141.1
H16C—C16—H16E	56.3	H36C—C36—H36E	56.3
H16D—C16—H16E	109.5	H36D—C36—H36E	109.5
C11—C16—H16F	109.5	C31—C36—H36F	109.5
H16A—C16—H16F	56.3	H36A—C36—H36F	56.3
H16B—C16—H16F	56.3	H36B—C36—H36F	56.3
H16C—C16—H16F	141.1	H36C—C36—H36F	141.1
H16D—C16—H16F	109.5	H36D—C36—H36F	109.5
H16E—C16—H16F	109.5	H36E—C36—H36F	109.5
C7—N1—C1	126.38 (10)	C27—N2—C21	127.45 (11)
C7—N1—H1N	116.8	C27—N2—H2N	116.3
C1—N1—H1N	116.8	C21—N2—H2N	116.3
			
C6—C1—C2—C3	2.1 (2)	C26—C21—C22—C23	−0.1 (2)
N1—C1—C2—C3	−177.99 (11)	N2—C21—C22—C23	−177.00 (12)
C1—C2—C3—C4	−0.5 (2)	C21—C22—C23—C24	0.3 (2)
C1—C2—C3—C14	178.23 (13)	C21—C22—C23—C34	−179.90 (14)
C2—C3—C4—C5	−1.4 (2)	C22—C23—C24—C25	−0.4 (2)
C14—C3—C4—C5	179.87 (14)	C34—C23—C24—C25	179.74 (15)
C2—C3—C4—C15	177.87 (14)	C22—C23—C24—C35	178.91 (14)
C14—C3—C4—C15	−0.8 (2)	C34—C23—C24—C35	−0.9 (2)
C3—C4—C5—C6	1.8 (2)	C23—C24—C25—C26	0.5 (2)
C15—C4—C5—C6	−177.47 (14)	C35—C24—C25—C26	−178.86 (15)
C4—C5—C6—C1	−0.3 (2)	C24—C25—C26—C21	−0.3 (2)
C2—C1—C6—C5	−1.7 (2)	C22—C21—C26—C25	0.2 (2)
N1—C1—C6—C5	178.37 (12)	N2—C21—C26—C25	176.86 (13)
O1—C7—C8—C9	21.3 (2)	O2—C27—C28—C33	148.70 (14)
N1—C7—C8—C9	−157.96 (13)	N2—C27—C28—C33	−31.60 (19)
O1—C7—C8—C13	−158.56 (14)	O2—C27—C28—C29	−30.15 (19)
N1—C7—C8—C13	22.2 (2)	N2—C27—C28—C29	149.55 (13)
C13—C8—C9—C10	−1.8 (2)	C33—C28—C29—C30	2.0 (2)
C7—C8—C9—C10	178.34 (13)	C27—C28—C29—C30	−179.12 (13)
C8—C9—C10—C11	1.6 (2)	C28—C29—C30—C31	−1.6 (2)
C9—C10—C11—C12	−0.2 (2)	C29—C30—C31—C32	0.0 (2)
C9—C10—C11—C16	178.81 (14)	C29—C30—C31—C36	−179.97 (14)
C10—C11—C12—C13	−1.0 (2)	C30—C31—C32—C33	1.4 (2)
C16—C11—C12—C13	180.00 (14)	C36—C31—C32—C33	−178.70 (16)
C11—C12—C13—C8	0.8 (2)	C31—C32—C33—C28	−1.0 (2)
C9—C8—C13—C12	0.6 (2)	C29—C28—C33—C32	−0.6 (2)
C7—C8—C13—C12	−179.55 (13)	C27—C28—C33—C32	−179.50 (13)
O1—C7—N1—C1	−1.9 (2)	O2—C27—N2—C21	0.1 (2)
C8—C7—N1—C1	177.33 (12)	C28—C27—N2—C21	−179.59 (12)
C6—C1—N1—C7	−148.38 (14)	C26—C21—N2—C27	27.1 (2)
C2—C1—N1—C7	31.7 (2)	C22—C21—N2—C27	−156.12 (13)

### Hydrogen-bond geometry (Å, °)

**Table:**

*D*—H···*A*	*D*—H	H···*A*	*D*···*A*	*D*—H···*A*
N1—H1N···O2^i^	0.86	2.29	3.0860 (14)	154
N2—H2N···O1^ii^	0.86	2.18	3.0101 (14)	162

Symmetry codes: (i) −*x*+1, −*y*, −*z*+1; (ii) −*x*+1, −*y*+1, −*z*+1.
